# SAR-Guided Scaffold Innovation of Selective V_2_R Antagonists: Therapeutic Frontiers in ADPKD

**DOI:** 10.1021/acsmedchemlett.6c00105

**Published:** 2026-03-31

**Authors:** Haofeng Shi, Yinlong Li, Steven H. Liang

**Affiliations:** † Department of Radiology and Imaging Sciences, 1371 Emory University, 1364 Clifton Road, Atlanta, Georgia 30322, United States; ‡ Wallace H. Coulter Department of Biomedical Engineering, Georgia Institute of Technology and Emory University, Atlanta, Georgia 30332, United States

**Keywords:** vasopressin V_2_ receptor (V_2_R), autosomal dominant polycystic kidney disease (ADPKD), G protein-coupled receptor (GPCR), structure−activity relationship (SAR), V_2_R antagonists

## Abstract

The vasopressin V_2_ receptor (V_2_R) is a class A G protein–coupled receptor (GPCR) that plays a pivotal role in the regulation of renal water homeostasis and has been implicated in the pathogenesis of autosomal dominant polycystic kidney disease (ADPKD) through sustained activation of cyclic adenosine monophosphate (cAMP) signaling. Pharmacological antagonism of V_2_R has emerged as a clinically validated strategy for attenuating cyst growth. However, the therapeutic application of currently available V_2_R antagonists remains constrained by safety liability and a relatively narrow chemical space. A recent study employed systematic structure–activity relationship (SAR) analyses to expand existing design paradigms for V_2_R antagonists, leading to the identification and synthesis of a series of structurally diverse V_2_R antagonist analogues. Supported by multidimensional pharmacological evaluations, these findings provide an important framework for the rational design and optimization of next-generation therapeutics for ADPKD.

## Graphical abstract

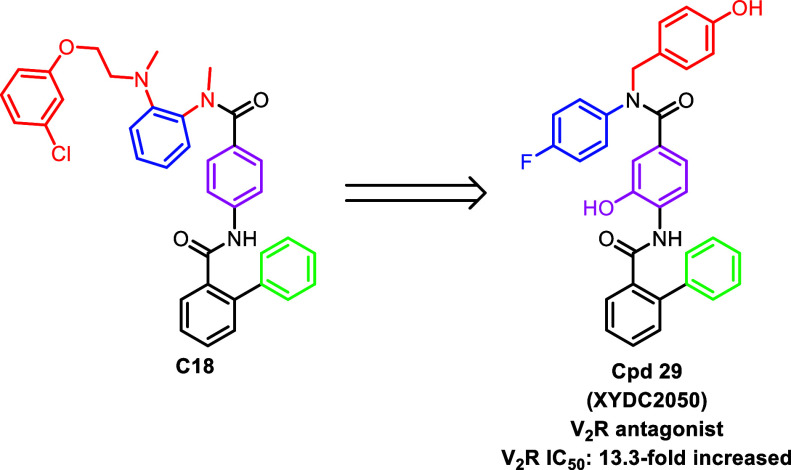

Autosomal dominant polycystic kidney disease (ADPKD) is among the most common monogenic kidney disorders and remains a leading cause of end-stage kidney disease.^1,2^ The ADPKD is driven by the persistent formation and progressive expansion of renal cysts, resulting in marked kidney enlargement and a gradual but irreversible decline in renal function over decades.^3,4^ Despite its substantial clinical and societal burden, therapeutic management has long been limited to supportive care and complication control.^5^ As a result, the development of disease-modifying, targeted therapies for ADPKD represents a critical unmet clinical need.^6^ In this context, growing mechanistic insights into the vasopressin-cAMP signaling axis have established the vasopressin V_2_ receptor (V_2_R) as a central regulator of cystogenesis and a clinically validated molecular target for therapeutic intervention in ADPKD.^7,8^

The V_2_R, a G protein-coupled receptor (GPCR) predominantly expressed on the basolateral membrane of renal collecting duct principal cells, is the primary mediator of arginine vasopressin signaling in the kidney.^9,10^ Converging mechanistic evidence has established sustained V_2_R-driven cAMP signaling as a central pathogenic axis in ADPKD, promoting cyst epithelial cell proliferation,^11^ dysregulated ion transport,^12^ and fluid secretion,^13^ thereby driving progressive cyst expansion,^14^ kidney enlargement,^15^ and functional decline.^16^ These insights have positioned V_2_R antagonism as a rational and clinically validated strategy for disease modification in ADPKD,^17^ exemplified by the approval of the nonpeptidic antagonist tolvaptan.^18^ However, the therapeutic potential of V_2_R inhibition remains incompletely realized due to limitations of existing agents, including suboptimal selectivity, hepatotoxicity risk, and mechanism-based aquaretic adverse effects,^19^ highlighting the need for next-generation V_2_R antagonists with improved safety and pharmacological profiles.

Conventional structure–activity relationship (SAR) studies of V_2_R antagonists have long assumed that a benzoazacyclic scaffold is indispensable for achieving high potency and receptor selectivity,^20^ which confines medicinal chemistry optimization to peripheral modifications and severely restricting the accessible chemical space.^21^ A recent study^22^ discoverd that high antagonistic activity and V_2_R subtype selectivity instead depend on effective occupation of a hydrophobic subpocket formed by three amino acid residues by an aromatic moiety, independent of the benzoazacyclic framework itself.^23^ Guided by this revised structure–function insight, the authors used the tolvaptan-derived compound **C18** as a starting point to design a series of V_2_R antagonists with nontraditional scaffolds.^24^

The nitrogen-containing seven-membered ring of **C18** was proposed to facilitate preorganization of the adjacent phenyl ring and orient it toward the hydrophobic subpocket. To expand scaffold diversity and assess whether this conformational constraint could be effectively replaced, the seven-membered ring was substituted with an ethoxy linker. As a result, compound **1** retained V_2_R binding affinity to a measurable extent. Subsequent optimization of linker length led to the identification of compound **3**, which exhibited improved binding affinity and was therefore selected as the lead scaffold for further optimization. Docking studies combined with SAR analysis suggested that rings a and b adopt relatively constrained and rigid conformations upon binding. Introduction of a *para*-fluoro substituent on ring a afforded compound **18**, which displayed the highest binding affinity, supporting the notion that incorporation of hydrophobic substituents to enhance hydrophobic subpocket occupancy in this region is advantageous. Binding mode analysis further revealed the presence of a positively charged side-chain residue in proximity to ring b, capable of acting as a hydrogen bond donor. Installation of a hydroxyl group at the 3-position of ring b (compound **22**) was hypothesized to exploit this potential hydrogen-bonding interaction, resulting in a further enhancement of V_2_R binding affinity. Additional binding mode analysis indicated that rings d and e of compound **22** were predominantly solvent-exposed, therefore, modifications at these positions were unlikely to disrupt key binding interactions but provided an opportunity to improve physicochemical properties such as aqueous solubility. Consistent with this rationale, introduction of polar substituents on ring e led to compound **29** (**XYDC2050**), which exhibited a marked improvement in water solubility while further optimizingV_2_R binding affinity (Figure 1).

**Figure 1:**
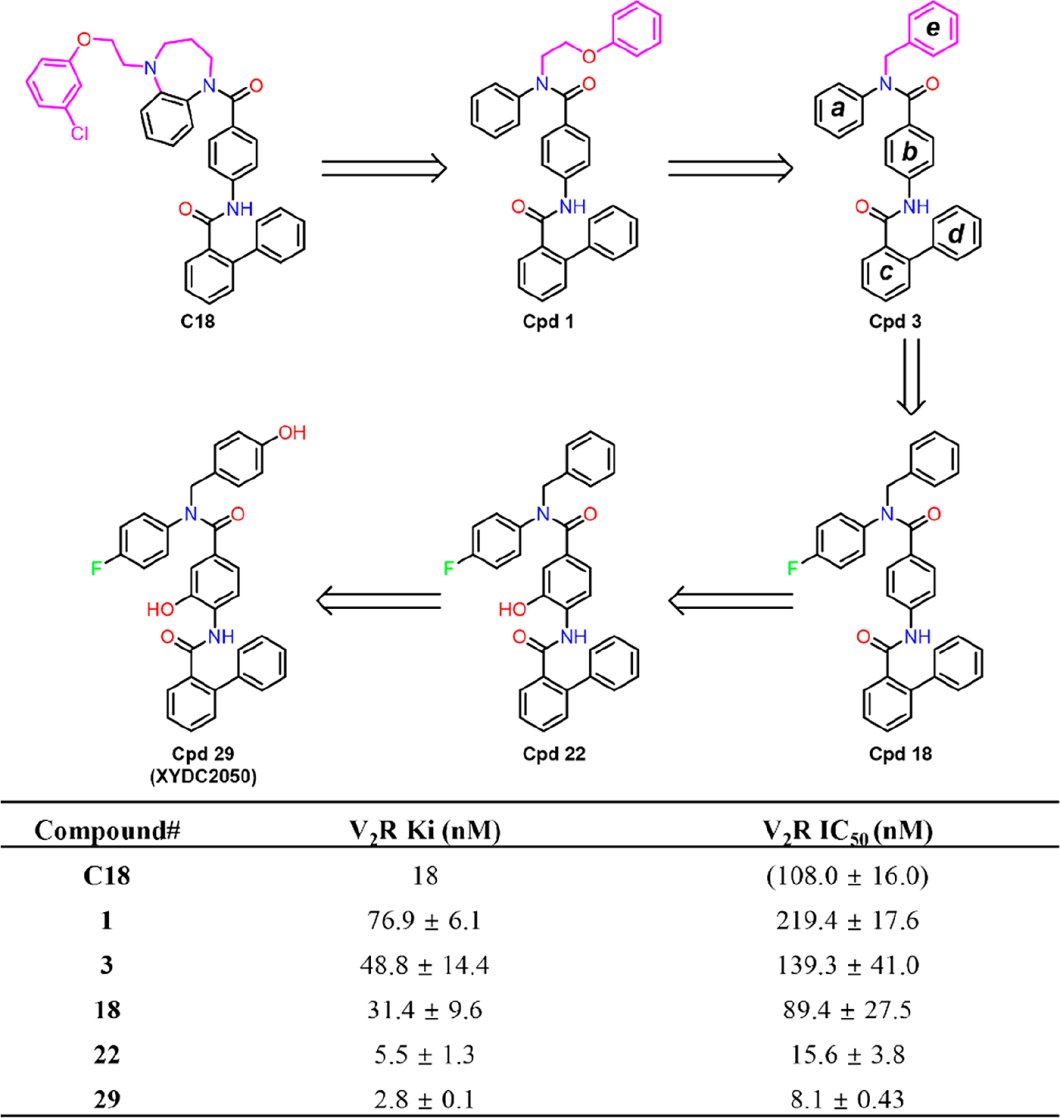
SAR optimization of compound **C18**. ^*a*^Data are mean ± SEM from 3 independent experiments with *K*_i_ values determined via fluorescent ligand displacement experiments. ^*b*^The IC_50_ values are determined from dose–response curves The data was adapted from ref 22. Copyright 2025 American Chemical Society.

Functional evaluation using cAMP assays identified that compound **29** potently inhibited vasopressin-induced cAMP accumulation, confirming its pronounced functional antagonism toward the V_2_R. Subtype selectivity profiling revealed that compound **29** exhibited a high degree of selectivity for V_2_R over V_1_aR, with a selectivity margin of 162-fold, which under the conditions of this study exceeded that of the clinically approved V_2_R antagonist tolvaptan (Figure 2, A). Pharmacokinetic characterization further indicated that compound **29** possessed a moderate elimination half-life and achieved peak plasma concentrations rapidly following administration, accompanied by a high maximum concentration and favorable systemic exposure, which provides adequate pharmacokinetic support for subsequent *in vitro* and *in vivo* pharmacodynamic investigations (Figure 2, B). In disease-relevant models, compound **29** markedly suppressed cyst growth in a dose-dependent manner in the Madin–Darby canine kidney (MDCK) cyst model (Figure 2, C). Consistent results were obtained in an *ex vivo* embryonic kidney model, further substantiating its inhibitory effects on cystogenesis (Figure 2, D). Moreover, *in vivo* evaluation in an ADPKD mice model revealed that treatment with compound **29** significantly reduced kidney enlargement relative to vehicle-treated controls (Figure 2, E). Given the known hepatotoxicity liabilities associated with tolvaptan, an additional safety assessment was conducted in wild-type C57BL/6 mice following 1 week of repeated dosing, which revealed no evidence of overt hepatotoxicity under the experimental conditions employed (Figure 2, F).

**Figure 2:**
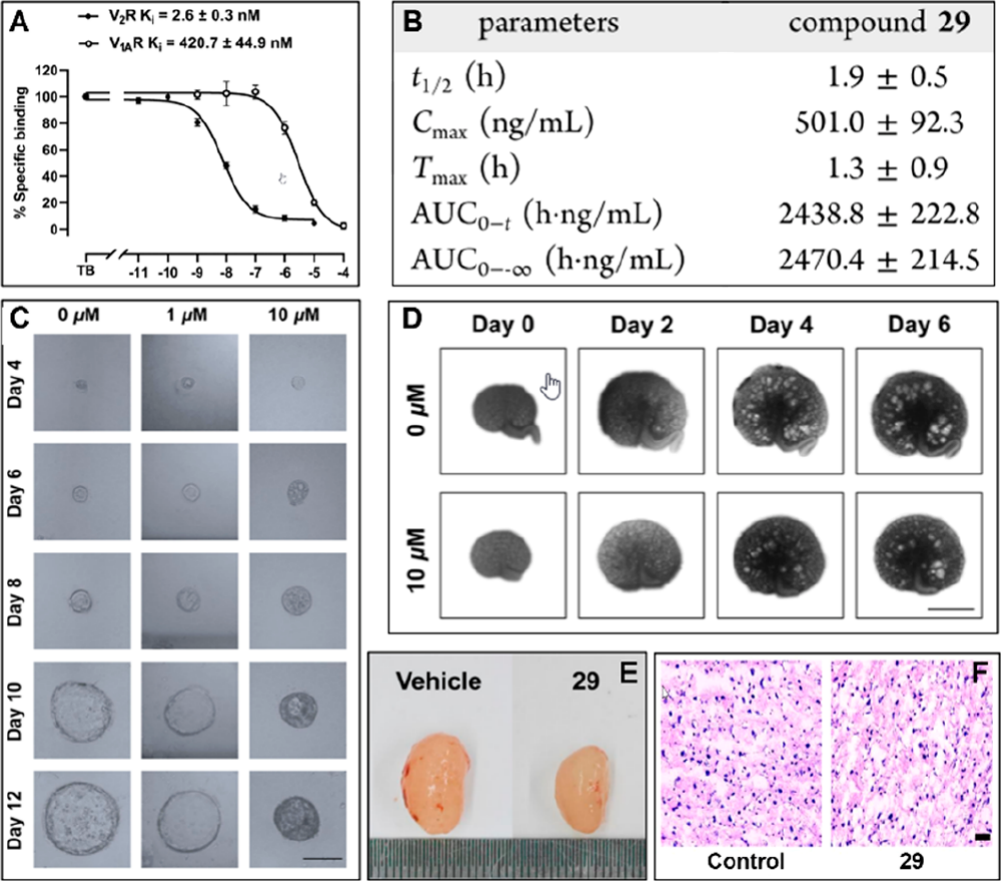
A. Receptor binding affinity and subtype selectivity of compound **29**; B. Key pharmacokinetic parameters of compound **29**. C. Dose-dependent suppression of cyst growth in an in vitro MDCK cyst model. D. Inhibition of cystogenesis in an ex vivo embryonic kidney model. E. Reduction of kidney enlargement in an ADPKD mouse model. F. Liver toxicity assessment of compound **29**. The data was adapted from ref 22. Copyright 2025 American Chemical Society.

## Future Outlook

Building on systematic scaffold optimization and extensive SAR investigations of V_2_R antagonists, compound **29** (**XYCD2050**) emerged from a series of analogues owing to its potent functional antagonism and high receptor affinity. Future studies should place more emphasis on long-term safety evaluations, complemented by comprehensive assessments of renal function parameters, to more rigorously validate its therapeutic potential in chronic settings. In addition, the development of radiolabeled V_2_R-targeting agents derived from compound **29** may enable positron emission tomography (PET)-based molecular imaging approaches for the noninvasive interrogation of V_2_R expression and regulatory dynamics during disease progression,^25,26^ thereby facilitating early monitoring of disease evolution and therapeutic response. Such imaging probes could allow quantitative assessment of in vivo target engagement, providing a powerful framework for refining dose–exposure–response relationships and optimizing dosing regimens.^27,28^

## Abbreviations

V_2_R: V_2_ receptor
GPCR: G protein-coupled receptor
ADPKD: Autosomal dominant polycystic kidney disease
cAMP: Cyclic adenosine monophosphate
SAR: Structure–activity relationship
SEM: Standard error of the mean
MDCK: Madin–Darby canine kidney
PET: Positron emission tomography.
